# Vision-based omnidirectional indoor robots for autonomous navigation and localization in manufacturing industry

**DOI:** 10.1016/j.heliyon.2024.e26042

**Published:** 2024-02-13

**Authors:** Cosimo Patruno, Vito Renò, Massimiliano Nitti, Nicola Mosca, Maria di Summa, Ettore Stella

**Affiliations:** Institute of Intelligent Industrial Technologies and Systems for Advanced Manufacturing, Italian National Research Council, STIIMA-CNR, Italy

**Keywords:** Omnidirectional autonomous robot, Visual odometry, Convolutional neural network, Computer vision, Feature-based approach, Manufacturing industry

## Abstract

In this paper, we present a new generation of omnidirectional automated guided vehicles (omniagv) used for transporting materials within a manufacturing factory with the ability to navigate autonomously and intelligently by interacting with the environment, including people and other entities. This robot has to be integrated into the operating environment without significant changes to the current facilities or heavy redefinitions of the logistics processes already running. For this purpose, different vision-based systems and advanced methods in mobile and cognitive robotics are developed and integrated. In this context, vision and perception are key factors. Different developed modules are in charge of supporting the robot during its navigation in the environment. Specifically, the localization module provides information about the robot pose by using visual odometry and wheel odometry systems. The obstacle avoidance module can detect obstacles and recognize some object classes for adaptive navigation. Finally, the tag detection module aids the robot during the picking phase of carts and provides information for global localization. The smart integration of vision and perception is paramount for effectively using the robot in the industrial context. Extensive qualitative and quantitative results prove the capability and effectiveness of the proposed AGV to navigate in the considered industrial environment.

## 1 Introduction

Mobile robotics is a hot topic where many researchers and pioneers are currently involved and are dedicating most of their efforts to achieve advances in this research field. Essential aspects of modern robotics are undoubtedly the accurate localization and the robust navigation for most mobile robot applications [1,2]. Over the recent decades, strong attention has been focused on developing novel technologies, methods, and solutions to achieve better localization and positioning of robots. Specifically, accurate localization increases the capabilities of autonomous systems, vehicles, and robots to navigate inside known and unknown environments. The success of missions or, similarly, the reduction of mission failures strongly depends on how the pose estimation is reliable and accurate.

The fourth industrial revolution, also called Industry 4.0, introduces new ideas and paradigms that are changing the way of conceiving robot navigation and localization [3,4]. Some of the areas primarily interested in this industrial revolution are logistics and transport. While in the past, it was accepted that the mobile robot had to operate inside a delimited area, nowadays this is perceived as a limit to overcome. In this regard, the mobile robots will work in different zones of warehouses, industrial plants, and factories to maintain high productivity and ergonomy and improve industrial processes. Consequently, this new generation of mobile robots needs the ability to navigate autonomously and intelligently by interacting with the environment, including the workers and other dynamic obstacles. Besides, reliable localization is mandatory and required [5]. Vision and perception are crucial elements in this context.

**Fig. 1:**
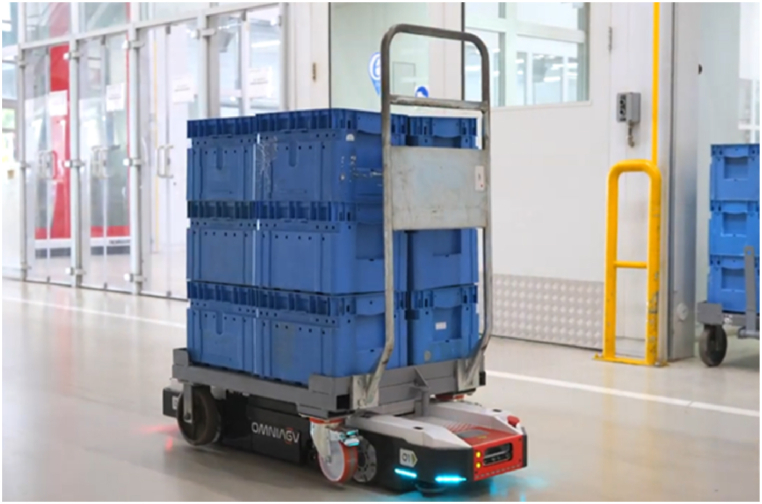
Prototype of the omnidirectional AGV carrying a cart in the manufacturing industry.

In this paper, we describe the prototype of an autonomous mobile robot (omniagv) able to move flexibly inside a manufacturing plant. This omnidirectional AGV can carry short carts containing raw or waste materials, more generally goods, as shown in Fig. 1.

As the AGV has to hook these short carts, it has to have a low height in order to move under the cart. This aspect involves low viewpoints to perceive the environment, which could hinder the correct navigation when classic approaches are used. Moreover, even if an industrial context could be potentially infrastructured, there is a high-growing demand for flexibility and reconfiguration of the environments that suggests using sensors directly on board the AGV, even for localization purposes. Additionally, the need to operate inside narrow spaces requires omnidirectional locomotion. Finally, the challenging and dynamic environment where the robot is expected to work can hamper the vision-based systems. In this regard, very low-textured surfaces (e.g., white walls or homogeneous floors), the presence of moving obstacles (see people, milkruns, etc.), and the requirement of avoiding the introduction of expensive changes to the infrastructures, have led to the development of robust and accurate vision-based systems for the adaptive robot navigation and localization in this context. An informative video about this AGV is available on YouTube [6].

In particular, the paper emphasizes the following aspects related to the sensing sub-system of the AGV, both considering specifications, design, and assembly of the robot and the implementation of the software modules controlling its behavior.

• Overall design of a system supporting localization and navigation tasks while fulfilling autonomous missions for transporting goods between specific origins and destinations, using sensing redundancy for enhancing its effectiveness;
• Hardware/software design of a downward-looking vision-based odometer taking advantage of the micro-textures present on the industrial floor and its smoothness for devising a practical localization solution;
• Sensing integration towards scene understanding, especially for object recognition and obstacle avoidance tasks;
• Docking solution for enabling the AGV of loading and unloading carts, either used to move goods or materials.

The overall approach in devising these objectives was to keep the environment in which the robot is required to operate almost unaltered, condensing all the sensing needs on the robot itself, with a single exception where simplicity and cost-effectiveness of external markers do not jeopardize flexibility, i.e. in docking operations while approaching carts.

The paper is organized as follows: Section II discusses the related works. Then, the description of developed vision-based modules is in Section III, whereas the explanation of experiments and their outcomes are reported in Section IV. Final conclusions and future works are in Section V.

## 2 Related works

Over the years, many AGVs have been developed according to the specific requirements of the market [7,8]. Some AGVs are still prototypes, whereas others are used in industrial plants. In this regard, several leading companies in the logistics field have started developing technologies and solutions, such as Swisslog Holding, CONVEYCO, fetch robotics, 6rive, Geekplus, AIUT Ldt., ABB, etc. For example, the ITU-AGV [9] is a ROS-enabled prototype developed by the ITU Robotics laboratory. This AGV is equipped with front and rear 2D laser scanners and one 3D lidar. A frontal camera and one inertial measurement unit are also used. It has two castor wheels and two differentially driven motor wheels. The navigation and localization system relies on a matching-based algorithm of 2D/3D lidar data to provide the robot pose in the environment. Passive and active cameras are not used for localization tasks. Another prototype of AGV (BoxAGV) is proposed in Ref. [10]. It is made of an aluminum frame with four meccanum wheels fastened. A frame camera looking towards the floor is installed. In this way, a predetermined route can be identified by the camera. Although this prototype can move omnidirectionally, it can navigate centrally by the LED stripes that define the paths. This involves some critical aspects such as: the inclusion of predefined paths into the plant; the need to keep the LED that is not always ensured inside an industrial environment; the need to power on the LED strips. Another study deals with autonomous mobile robotics (AMR) technology for smart factories [11]. In this case, the mobile robot MIR100 [12] has been used to cooperate within the production line by taking the manufactured products and then delivering them to the areas of interest. This AGV is equipped with two SICK S300 laser sensors placed at opposite corners to avoid any blind spots around the robot. The limitations of these sensors reside in the limited field of view for detecting obstacles higher than 200 mm and the incapability to manage transparent objects or reflective surfaces that might involve data inaccuracies. Some of these limitations are overcome by using 3D cameras. However, only one stereovision system is fastened at the front of the robot, thus dramatically limiting the field of view. In this regard, this sensor cannot detect other obstacles located at the robot sides. Four ultrasonic sensors complete the hardware. The locomotion system comprises two differential wheels, whereas four swivel wheels are used to ensure stability during the motion. Other applications that exploit the MIR platform are reported in Refs. [13,14].

Another logistics system (Smoov ASRV) employs moving platforms to handle pallets in a structured smart warehouse [15,16]. The AGV uses inductive sensors, one vision system based on laser profilometry, and wheel encoders for estimating its pose. However, the robot is constrained in moving inside the predefined paths of metallic rails. It owns four wheels that can rotate to allow the robot to change the movement direction.

Other solutions require opportunely structuring the environments, as in the logistics system adopted by the Amazon company [8,17]. In this regard, the Kiva robot can retrieve movable shelf racks by navigating the warehouse through a series of QR codes fastened on the floor. Each drive unit has proximity sensors that prevent fatal collisions with others. The robot has differential wheels and can move by performing pure rotations on spot and orthogonal translations. The fact that the robot can follow only specific paths heavily limits its operability and flexibility in industrial contexts or inside environments where the facilities cannot be remodeled or structured with fiducial markers. ABB proposed a similar solution that ensures maximum agility, which proposed a family of moving platforms called Flexley Mover AMRs [18]. The AGV uses two safety laser scanners for detecting obstacles and exploits the QR codes for visual navigation. In this case, the robot can move omnidirectionally, providing excellent maneuverability. However, the environments need to be equipped with artificial markers, with the drawback that a periodical redefinition of the infrastructures involves changes in the marker locations, thus hampering the flexibility of the system.

Table 1 compares the previously described AGV systems, highlighting the main features of each. The analysis of the literature reveals that many devised AGVs or AMRs rely on external artificial markers for navigating into an industrial plant [8,10,17,18]. Moreover, using some families of robots could require a drastic redefinition of the facilities. Most robots have limited moving capabilities that can negatively affect operations when narrow spaces need to be managed [8,10,15] [[8], [10], [15], [16], [17]] [[8], [10], [15], [16], [17]]. By comparing the proposed solution with the other ones, it can be noticed that some AGVs are still prototypes [9,10,15,16]. Among the off-the-shelf systems [8,11,17], two of them [8,17] rely on heavily structured environments. The work in Ref. [11] seems to be the most similar solution to our proposal, but it uses differential wheels for making movements that hinder maneuverability in narrow spaces. Last but not least, the [11] has a limited field of view of the environment.

**Table 1:** Taxonomy of proposed AGVs described in Literature. The last row reports our proposal. The external features row indicates whether the system or the AGV uses external markers for some tasks.

	Locomotion	Application	Hardware	Software	Payload	Environment	External features	Availability
ITU-AGV [9]	Two differential wheels	Logistics Manufacturing industry	3D lidar 2D lidar IMU	ROS	Unknown	Unstructured	No	Prototype
BoxAGV [10]	Four omnidirectional wheels	Logistics	2D Camera	Unknown	Unknown	Structured	Yes	Prototype
MiR100 [11]	Two differential wheels Four swivel wheels	Smart factory	SICK S300 scanners 3D cameras (Intel RealSense™) Ultrasonic sensors IMU Wheel encoders	Unknown	100 kg	Unstructured	No	Ready-made solution
Smoov ASRV [15,16]	Four rotating wheels	Smart warehouse Logistics	Inductive sensors Wheel encoders Vision-based system	C++ Siemens Step 7	150 kg	Structured	No	Prototype
Amazon Kiva Robot [8,17]	Differential wheels	Logistics	Proximity sensors Two cameras (for barcodes on floor and on racks) Infrared sensors Collision-detection bumpers	Unknown	450 kg	Structured	Yes	Ready-made solution
Flexley Mover AMRs [18]	Omnidirectional wheels	Storage operations Logistics Material handling	Magnetic sensors Two laser scanners	Unknown	600 kg	Structured	Yes	Ready-made solution
**Proposed OmniAGV**	Omnidirectional wheels	Logistics Material handling	Two laser scanners Four stereocameras One optical encoder Wheel encoders Proximity sensors IMU	C++ Python ROS	350 kg	Unstructured	Yes	Ready-made solution

This paper proposes an omnidirectional AGV able to carry carts into a manufacturing warehouse. Differently from the already proposed solutions, this one has been devised to keep the infrastructures unaltered and move naturally into the environment by managing possible moving and stationary obstacles. The requirements are.

• The AGV has to hook very short carts. Moreover, a low AGV is needed to move under the carts.
• The AGV has to move inside very narrow spaces, such as the supply and storage lanes.
• The carts and the environments cannot be heavily adapted to the AGV. The AGV has to fit the facilities of this use case.
• The AGV needs to be highly maneuverable and agile. Consequently, a new concept of omnidirectional wheels was devised to meet this requirement.
• The industrial environment where the robot works is very challenging for vision-based systems. Therefore, hardware and software integration have been pushed to develop robust and intelligent vision-based modules.

The proposed system exploits proprioceptive sensors, such as laser scanners, wheel odometers, vision-based odometers, stereocameras, and IMU, to naturally navigate inside very dynamic, unstructured, and challenging environments. External markers are used sparingly, where their simplicity and cost effectiveness do not jeopardize flexibility. Indeed, they are easily applicable to carts and strategic points of the facility and are used for cart detection and global localization purposes. No major redefinitions and modifications of the carts and the facilities are needed.

## 3 Approach

**Fig. 2:**
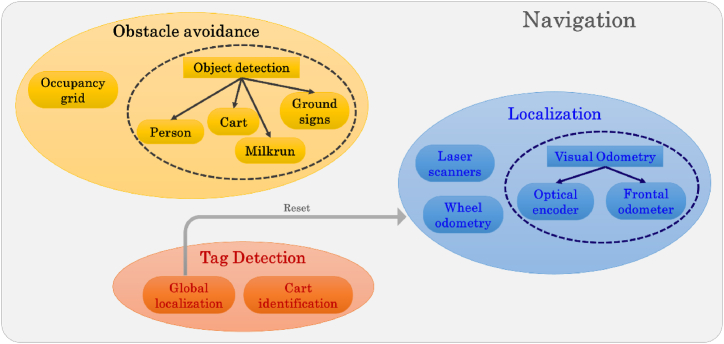
Overview of the main modules used by the AGV. The *Navigation* module is composed of three macro modules: Localization, Obstacle avoidance, and Tag detection. All these modules are helpful for the autonomous navigation and localization of the AGV into the industrial environment.

This section describes the main modules integrated into the mobile robot developed for logistics in the factory. As shown in Fig. 2, the *Navigation* module includes three macro modules.

• *Localization*
• *Obstacle avoidance*
• *Tag detection.*

The *localization* module provides all the signals and commands to localize the AGV into the industrial environment. In detail, it uses laser scanners, wheel odometry, and visual odometry to estimate its relative and absolute pose. The *obstacle avoidance* module detects all the obstacles that might hinder the robot during its mission into the plant. Specifically, this module comprises two inner separated modules: *Occupancy grid* and *Object detection*. The *object detection* module can recognize different object classes, e.g. people, carts, milkruns, ground signs, etc … Then, adaptive behaviors can be implemented according to the belonging class of the detected object. The *occupancy grid* module generates a dynamic occupancy map used for navigating the robot into the environment adaptively. Finally, the *tag detection* module enables the recognition of fiducial markers. It has a twofold use: it can be used for resetting the odometry systems, thus updating the absolute pose (*Global localization* module), and identifying the carts and their middle hanging points (*Cart identification* module).

**Fig. 3:**
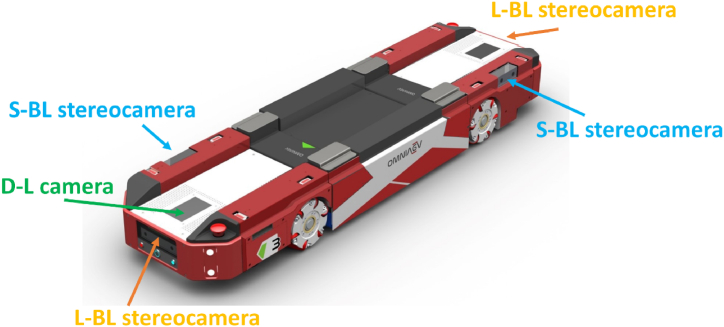
Perception systems used by the developed mobile robot. Monocular cameras and stereocameras (short and long baselines) are fastened at the front and rear of the AGV to provide the information handled by the modules.

Different perception systems are integrated into the AGV, as shown in Fig. 3. Four stereocameras are mounted around the mobile robot, where two of them are located at the front and the other two at the rear. One monocular camera, oriented toward the floor, is also placed on the front side of the robot. All these vision sensors enable sensing the environment, and the previously described modules provide all the valuable information for autonomous robot navigation and localization. The following sections will explain in detail each of the modules listed.

### 3.1 Localization module

**Fig. 4:**
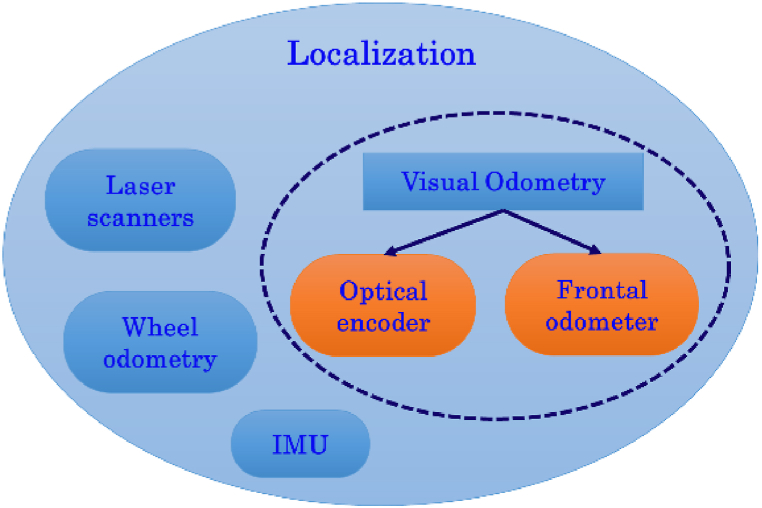
Overview of localization module. The visual odometry system uses the frontal stereocamera and the D-L camera (see orange shapes).

The *localization* module provides the robot pose using common sensors such as laser scanners, inertial measurement units (IMUs), and passive monocular and stereo cameras (see Fig. 4). As the environment where the robot works is really challenging, merging multiple signals to enhance the robustness and accuracy of the information provided to the navigation module is crucial. Constraints related to the floor, walls, and the low height of the robot that involves reduced viewpoints, affect the data returned by the sensors. For example, wheel slippages introduce drift errors in the wheel odometry, or textureless and high-reflective surfaces as white walls or homogenous floors can negatively affect the visual odometry systems. Consequently, signals returned from different sensors undoubtedly help improve the robot's pose estimation. The basic idea is that one or more sensors can act as effective failover strategies for environmental sensing and localization if another data source is missing or is recognized as corrupted.

The visual odometry uses two passive cameras: the downward-looking (D-L) camera and one forwarding-looking stereocamera with a long baseline (L-BL). Both the odometers use the features-based approach. The extracted keypoints from the flow of images are related to each other over consecutive frames using reliable and robust descriptors. Nevertheless, the matching phase often provides incorrect associations of keypoints mainly due to the low-textured environment, as the considered one, where the AGV works. The proposed methods effectively reject the incorrect correspondences, thus enhancing the relative pose estimation.

Section 3.1.1 will describe all the methods related to the downward-looking odometer, which essentially exploits the 2D data for extracting the information of interest, whereas Section 3.1.2 is mainly associated with the forward-looking odometer that uses both the 2D and the 3D data for the ego-motion estimation.

#### 3.1.1 Downward-looking vision-based odometer

The downward-looking vision-based odometer (also referred to as the optical encoder in the manuscript) is designed to estimate the relative motion of the robot by analyzing directly the floor images. A monocular vision system, having its optical axis nearly perpendicular to the floor, is used. The following paragraphs exhaustively describe the adopted lighting system and explain the processing steps for this vision-based odometer.

##### 3.1.1.1 Lighting system

The particular industrial environment wherein the robot has to work is very challenging for vision-based approaches. The high reflectivity of the floor together with its low textureness, yield the motion estimation harder. Consequently, besides developing a robust method for deriving motion information, a specific lighting system must be designed for these contexts.

**Fig. 5:**
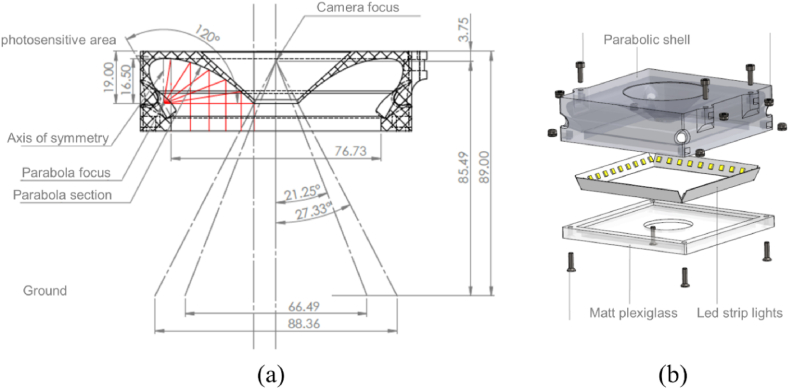
(a) Side view of the designed paraboloid shell. (b) A comprehensive lighting system made of the parabolic shell, the led strip lights, and the matt plexiglass used for uniformly spreading the light beam. Made available by courtesy of Code Architects Automation Srl.

As the floor is prevalently uniform in terms of appearance and the textured patterns occasionally occur, an illumination system that better emphasizes the floor microstructures, or the less visible appearance features, is needed. Inadequate lighting can be counteracted by longer exposition times or digital gain, but ultimately they affect image quality, for example inducing blur effects, thus negatively impacting the final motion estimation. Moreover, an indirect lighting approach, rather than direct lighting, must also be considered to avoid introducing light spots into the images, which might produce apparent motion of the features. All these considerations have brought to designing a specific lighting system, as reported in Fig. 5.

This lighting system can produce uniform, coherent, and indirect vertical illumination of the floor. A parabolic shell for the illuminator is devised to ensure the required properties. Specifically, the interesting reflective property of the parabola has been taken into account [19]. It asserts that in case a light source is placed at the focus of the paraboloid, the light beam is reflected outward along the direction of the axis of symmetry (please refer to Fig. 5(a)). The half sections of parabolas enable to spread the light beam perpendicularly toward the floor. The flexible led strip lights are mounted in correspondence with the foci of paraboloids. A plexiglass panel, opportunely dulled and in charge of spreading and making homogenous the output light beam, completes the lighting system (see Fig. 5(b)). The camera is housed in the middle of the illumination system, which has been built using a 3D printer.

##### 3.1.1.2 Keypoints extraction and rectification

**Fig. 6:**
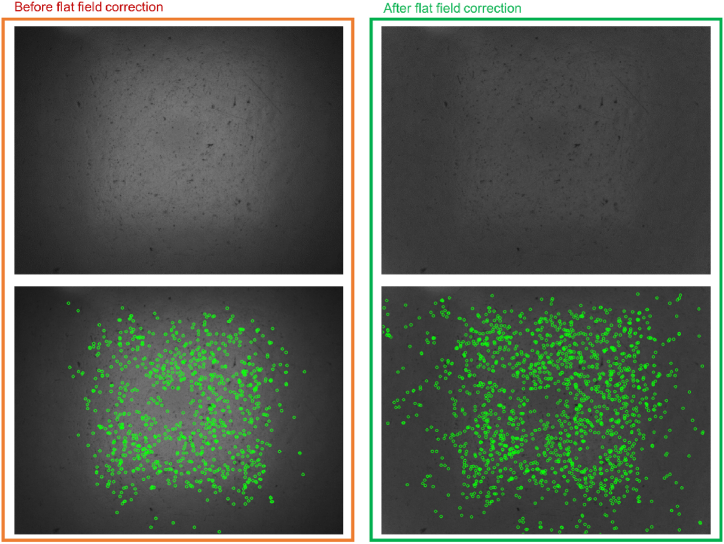
In the first column (red rectangle), the input image (top) and the corresponding keypoints (bottom) extracted before the application of the flat field correction, are shown. After the FFC, the intensity values of pixels are adjusted (green rectangle). This causes a better spreading of the visual keypoints (bottom).

Before extracting the keypoints from an input image, it is mandatory to perform some preliminary processing to enhance the image quality in terms of the intensity value distribution of pixels. In this regard, the flat field correction (FFC) is commonly used to compensate for the different gains and the dark currents in the photosensitive camera sensor that yield the image borders to be less bright than the middle area, also known as the vignetting effect [20]. Without this precautionary correction, the keypoint distribution would be primarily located in the middle of the image, as reported in Fig. 6.

A non-uniform keypoints distribution would involve less accurate motion estimation, especially when the AGV, on which the vision-based odometer is fastened, is performing rotational movements. After the FFC is applied, the keypoints are better located over the entire image, as clearly shown in the second column of Fig. 6. In this way, also appearance features that might be found at the margin of the image, are better highlighted and then taken into account by the subsequent processing stages.

The FFC is performed by imaging a uniformly illuminated area, thus producing an image of uniform color and brightness. By using Eq. (1), it is possible to correct the input image I properly.

(1) $$C=\frac{\left(I-D\right)m}{\left(F-D\right)}$$

The matrix *D* represents the dark field, or the dark image, obtained without incident light. It is usually acquired by blinding the camera lens with the shutter. The matrix *F* represents the flat-field image gathered when the camera frames a uniformly white color panel. The scalar *m* is the average value of the matrix (*F*-*D*).

After the preliminary processing of the image, the keypoints of interest can be detected. The downward-looking vision-based odometer uses the speeded-up robust features (SURF) [21] approach to detect distinctive, invariant, and robust 2D points locally. Inspired by the scale-invariant feature transform (SIFT) [22], SURF performs much better than its counterpart in terms of time performance and robustness against noise and different image transformations such as rotations and warping.

Besides the correct matching between keypoints over consecutive frames, it is worth rectifying the detected points. In fact, the images are affected by unwanted distortion effects mainly due to the imaging setup that can change the perceived distance between feature points. Neglecting this aspect will involve higher reprojection errors during the motion estimation of the robot. Therefore, the estimation of intrinsic and extrinsic camera parameters is mandatory for achieving better relative pose measurements. The unknown parameters can be estimated through conventional methods, as in Refs. [23,24], that exploit a known chessboard pattern acquired by the camera under different poses.

**Fig. 7:**
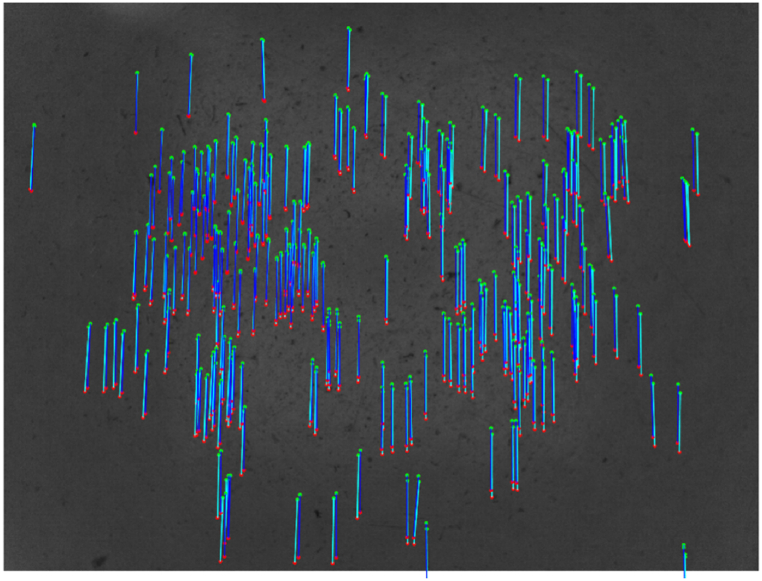
Keypoints in the target image (red dots), source image (green dots), and correspondences before (cyan lines) and after (blue lines) the rectification step. The rectification of keypoints is mandatory for achieving better motion estimates.

As already stated, the optical axis of the camera is nearly perpendicular to the floor plane. Starting from the assumption that the AGV does not have suspension systems, the camera height from the floor never varies. Consequently, the scale factor is fixed and can be computed a priori. However, slight tilting of the image plane with respect to the floor plane (i.e., the two planes are not perfectly coplanar) yields different scale factors across the entire image, although of small margins. Suppose the camera tilt with respect to the floor plane is not opportunely compensated. In that case, the measurements will be affected by increasing errors that progressively accumulate over time, thus leading to worse estimates. Similarly to Ref. [25], the alignment calibration step has been performed to mitigate these unwanted errors. The effect of these compensations is qualitatively evident in Fig. 7.

The red dots represent the detected keypoints in the target image (or previous image). In contrast, the green dots are the same keypoints located in the source image (or current image) after the robot motion. The SURF keypoints over consecutive images are related to each other through the SURF descriptors. The matches between keypoints are represented utilizing lines (optical flow representation). The cyan lines refer to the matches without applying the aforementioned point rectification, whereas the blue lines stand for the rectified matches. One can notice that the correspondences are slightly mislocated due to the rectification effect. This misalignment involves different motion estimations for the considered pair of images. Quantitative analysis for the considerate example has revealed that these differences in motion estimation are equal to about 0.1 mm for translation and 0.08 deg for the rotation. Although the differences are low, they cannot be neglected if more accurate measurements are expected.

##### 3.1.1.3 Correspondences statistical filtering

Despite the SURF approach being efficient for most CV-based and VO-based applications, sometimes the environment typology, as the considered one, might negatively affect the robust correlation among the keypoints over the images. Repetitive patterns and low-textured surfaces might produce similar SURF descriptors, thus implying incorrect matches. The downward-looking vision-based odometer implements statistical filtering to reject false matches.

**Fig. 8:**
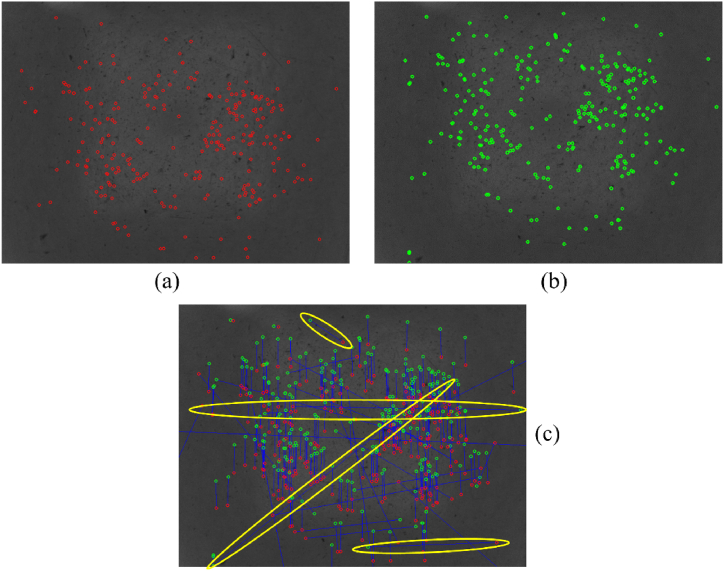
(a) Previous keypoints (red dots) in the target image, (b) current kepypoints (green dots) in the source image, and (c) correspondences between the keypoints superimposed on the source image. Some incorrect matches to be removed are in the yellow marks.

Fig. 8(a–b) reports the detected keypoints for two consecutive images and their related matches according to the SURF descriptors. In Fig. 8(c), yellow marks highlight some of the incorrect correspondences.

To discard those incorrect 2D point associations, a histogram analysis of correspondences is performed. An efficient method has been proposed in Ref. [26] for the outlier removal. As this method assumes some simplifications that are met in case of slight angular and translational variations, we developed our method that works on the Euclidean distance and the orientation without making any simplifications that are not more negligible in case of large motions. Hence, the correspondences are evaluated in terms of their orientation and magnitude. Starting from the assumption that, after the robot motion, the correspondences have to be coherent across the whole surface, it is expected that the obtained histograms should be comparable to a Gaussian distribution having a low standard deviation. Therefore, the outliers should be located at the margin of the distribution, whereas the inliers should be mainly situated near the average value. Consequently, statistical information such as the average value and the standard deviation are used to detect the outliers.

**Fig. 9:**
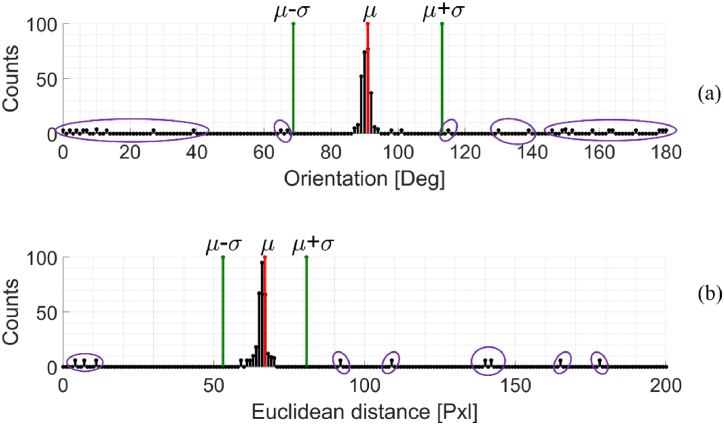
(a) Orientation histogram and (b) Euclidean distance histogram computed of the feature correspondences for the case under investigation. The extraction of statistical information related to the two histograms enables to filter out the outlier matches efficiently. The variables μ and σ represent the average value and the standard deviation referred to the considered distribution. The peaks in the purple marks represent the outliers.

Fig. 9 reports the obtained histograms for the case under investigation. All the orientations of the joint straight lines are cumulated into 180 equally-spaced bins (Fig. 9(a)). Each bin covers one degree. The peak of this distribution indicates the dominant orientation of correspondences. The peaks out of the statistics are considered as outliers.

Similarly, the histogram of the Euclidian distances related to the correspondences is derived (Fig. 9(b)). These Euclidean distances, expressed in pixel units, are cumulated into 200 bins. In this case, the number of bins has been opportunely set by taking into account the following consideration: supposing that the keypoint has to be visible at least across three consecutive frames for ensuring an adequate feature overlapping, one-third of image height is used as bin number. The motion could not be estimated if there was not enough overlapping between keypoints across frames.

At this stage, all the correspondences that do not meet the statistical requirements of both distance and orientation, i.e. those having values out of the range {μ−kσ,μ+kσ}, where μ and σ stand respectively for the average value and the standard deviation of the specific distribution, are discarded. The scalar *k* represents the strength of the filter. In this work, it has been empirically set to 1.

**Fig. 10:**
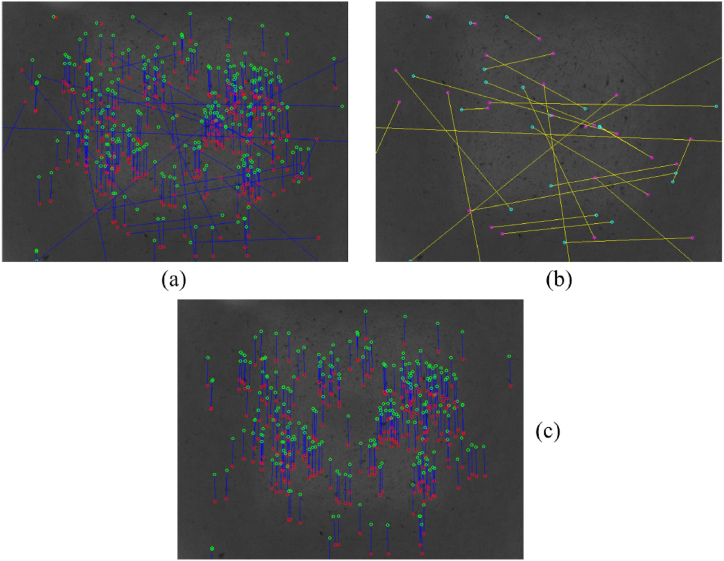
Feature correspondences (a) before and (c) after applying the statistical filtering. (b) Detected outliers that remarkably differ from the inliers.

Fig. 10(a) shows the starting correspondences to be processed employing the proposed statistical filtering. The detected outliers are reported in Fig. 10(b). As can be noticed, the outliers considerably differ from the final inliers reported in Fig. 10(c). The ultimate correspondences are all coherent in terms of orientation and magnitude, thus proving the effectiveness of the proposed approach. These correspondences are then used for estimating the relative robot pose, as described in Section 3.1.3. The pseudocode of the procedure related to the downward-looking vision-based odometer is reported in Algorithm 1.

**Figure:**
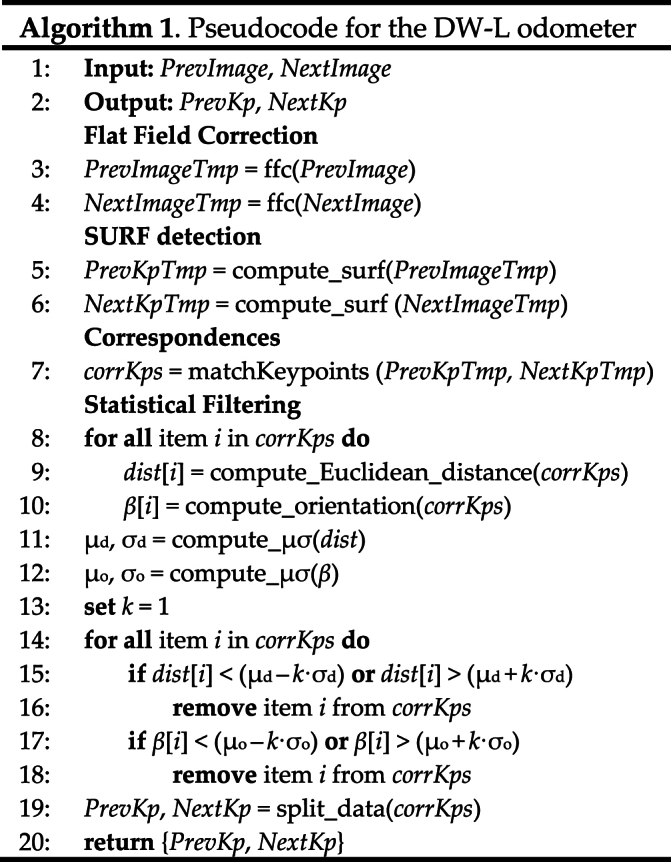

#### 3.1.2 Forward-looking vision-based odometer

The forward-looking vision-based odometer (also named frontal odometer in this manuscript) exploits the left passive camera of the stereovision system for estimating the AGV pose. Similarly to the optical encoder, the visual odometry approach processes consecutive images from the left camera. The role of the frontal odometer is to provide redundant information about the robot motion by still using vision perception to implement the aforementioned failover strategy. For example, when the optical encoder does not provide any pose estimation, the frontal odometer can compensate for this lack and vice versa. In fact, the challenging environment where the robot moves can heavily hinder the relative pose estimation by the two vision-based odometers when some critical situations occur. The redundancy of pose estimation by vision increases the accuracy and robustness of the relative localization.

The frontal odometer also takes advantage of the 3D information returned by the stereovision system. The feature-based approach is also used in this case. However, the oriented FAST and rotated BRIEF (ORB) detector [27] is used to extract the keypoints from the image flow. Some experimental tests have proven that ORB performed better than SURF for frontal odometry. Specifically, ORB seems to be more robust to illumination and rotational changes. In the case of the optical encoder, the illumination is homogeneous, and it is not affected by external lights because the sensor is located at the bottom of the AGV, which acts as a shield for light. On the contrary, the frontal odometer is affected by external lights that cannot be neglected. As a consequence, ORB has been preferred instead of SURF.

**Fig. 11:**
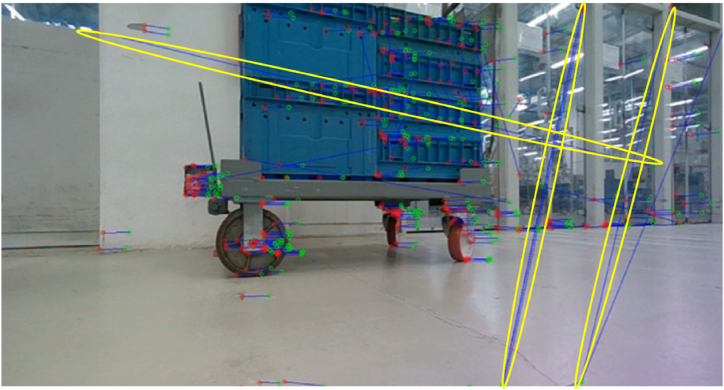
The detected ORB keypoints in the target image (red) and source image (green) superimposed over the source image. Some incorrect matches are highlighted in yellow marks.

An example of detected ORB keypoints over two consecutive images is shown in Fig. 11. The keypoints of the source image are in green, whereas the keypoints of the target images are in red. Blue segments represent the correspondences using the BRIEF descriptor.

The 2D keypoints are rectified from distortion effects as made for the optical encoder to ensure better pose estimates. Then, for each 2D keypoint, it is possible to extract the z-coordinate from the depth map returned by the stereocamera. In this way, the 3D location of the keypoint can be associated. At this stage, a RANSAC-based method [28] is used to remove outliers that produce incorrect correspondences (see yellow ellipses in Fig. 11). These wrong matches must be removed to increase pose estimation accuracy. After the coarse pose estimation (see Section 3.1.3 for details), a refinement algorithm based on Levemberg-Marquardt [29] solver was implemented, thus ensuring more accurate estimates.

The related pseudocode is reported in Algorithm 2 as follows.

**Figure:**
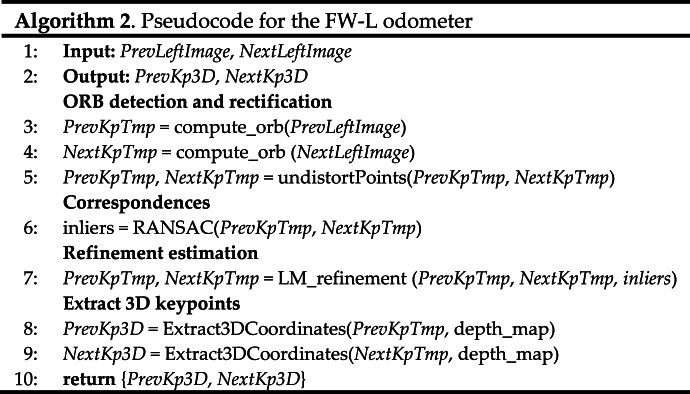

#### 3.1.3 Ego-motion estimation

This section describes how the relative pose of the robot is estimated for the two VO-based systems. The keypoints, opportunely filtered to remove incorrect correspondences, are converted into metric units by using the intrinsic and the extrinsic camera matrices. This conversion is straightforward for the frontal odometer because the stereocamera also returns the depth map.

At this stage, two sets of 3D keypoints are used to assess the motion between two consecutive images by solving a non-linear system of equations. Let $k_{S}$ and $k_{T}$ be the list of source keypoints and target keypoints, respectively, it is possible to estimate the information about the relative motion $\left(t_{x},t_{y},t_{z},\alpha ,\beta ,\gamma \right)$ encoded in the *4x4* affine matrix A by using Eq. (2).

(2) $$k_{T}=A\cdot k_{S}=\left[\begin{array}{c} x_{T}\\ y_{T}\\ z_{T}\\ 1 \end{array}\right]=\left[\begin{array}{cccc} \cos\;\alpha \cdot \cos\;\beta & \cos\;\alpha \cdot \sin\;\beta \cdot \sin\;\gamma -\sin\;\alpha \cdot \cos\;\gamma & \cos\;\alpha \cdot \sin\;\beta \cdot \cos\;\gamma +\sin\;\alpha \cdot \sin\;\gamma & t_{x}\\ \sin\;\alpha \cdot \cos\;\beta & \sin\;\alpha \cdot \sin\;\beta \cdot \sin\;\gamma +\cos\;\alpha \cdot \cos\;\gamma & \sin\;\alpha \cdot \sin\;\beta \cdot \cos\;\gamma -\cos\;\alpha \cdot \sin\;\gamma & t_{y}\\ -\sin\;\beta & \cos\;\beta \cdot \sin\;\gamma & \cos\;\beta \cdot \cos\;\gamma & t_{z}\\ 0 & 0 & 0 & 1 \end{array}\right]\cdot \left[\begin{array}{c} x_{S}\\ y_{S}\\ z_{S}\\ 1 \end{array}\right]$$

Hence, an affine transformation matrix $A_{i}$ can be computed from each pair of images. By composing the affine matrices over time by exploiting Eq. (3), it is possible to recover the last pose of robot encoded into the matrix $A_{n}$ and thus reconstruct the motion trajectory.

(3) $$A_{n}=A_{0}\cdot A_{1}\cdot A_{2}\ldots \cdot A_{i-1}\cdot A_{i}\ldots \cdot A_{n-1}$$

### 3.2 Obstacle avoidance module

The *obstacle avoidance* module provides all the proper signals and data for attaining obstacle avoidance by the robot during the navigation into the plant. This module is composed of two submodules: Occupancy grid and Object detection. The former provides a bi-dimensional map of the obstacle generated by processing 3D data. The latter is in charge of recognizing specific objects and returning the 3D location of these obstacles. The *obstacle avoidance* module is schematized in Fig. 2.

#### 3.2.1 Occupancy grid computation

**Fig. 12:**
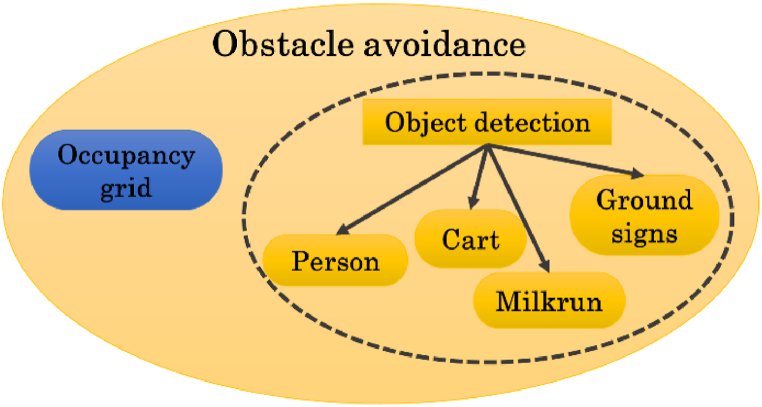
Scheme of the obstacle avoidance module. The occupancy grid module is highlighted in blue tone.

This paragraph describes the occupancy grid module belonging to the *obstacle avoidance* module, as shown in Fig. 12. Starting from the 3D data returned by one stereocamera, this module provides an occupancy map of all the moving and stationary obstacles that the sensor has perceived.

Being the AGV equipped with four stereocameras, having short and long baselines (two S-BL stereocameras and two L-BL stereocameras), the module returns back to the *navigation* module four occupancy grids. In this way, the robot knows about the obstacles around itself. As the robot is omnidirectional, obstacles at the sides of AGV have to be detected and managed to avoid fatal collisions. This motivates the use of lateral stereocameras. Also, the laser scanners placed under the AGV generate occupancy maps. However, the laser scanner sensors cannot detect floating or hanging obstacles. Consequently, the proposed vision-based systems are also mandatory for detecting these obstacles.

**Fig. 13:**
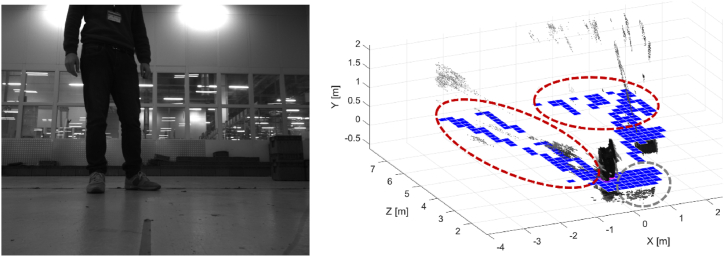
3D reconstruction of the scene reported on the left. The blue squares represent the occupancy grid obtained by using the point cloud without applying any filtering. The points in the red ellipses are due to incorrect projections of object borders in the 3D space. The points in the gray circle are due to the light reflections on the floor.

Using the methods described in Ref. [30], a robust 2D occupancy map useful for robot navigation tasks can be computed. The stereocamera produces a 3D reconstruction of the perceived environment. Nevertheless, the point clouds are noisy and present many outliers to be removed effectively. As an example, a 3D reconstruction of a scene is reported in Fig. 13. By using a predefined 2D grid, it is possible to estimate the density of 3D points belonging to that bin of the sampling grid. If one bin contains many 3D points, the color tends to be pink, whereas it remains blue. Looking at Fig. 13, the occupancy map is represented using a blue-to-pink color space. As can be observed, false obstacles are introduced into the occupancy map when the point cloud is not adequately filtered. The 3D points inside the gray mark are mainly due to unwanted reflections of light on the floor that involve incorrect reconstructions. Besides, the points inside the red marks are likely due to incorrect reconstruction of object borders. In this regard, the stereo matching algorithm might have produced false mismatches to discard. Finally, the objects in the background are not reconstructed because they are too far from the acquiring sensor.

By using a cascade filtering procedure, it is possible to discard all the unstable or unwanted 3D points. After a preliminary processing of the point cloud, a single-cell analysis is performed to remove noisy points while still preserving hanging objects. Different checks, such as spread check, density check, and noisy check, have been added to accomplish this task. The mathematical formulation related to these methods is reported in Eqs. (4), (5), (6).

(4) $$\begin{array}{ccc} X_{L}\leq x\leq X_{H} & Y_{L}\leq y\leq Y_{H} & \begin{array}{cc} Z_{L}\leq z\leq Z_{H} & I_{L}\leq i\leq I_{H} \end{array} \end{array}$$

(5) $$\begin{array}{cc} y\geq r_{yz}\cdot \sin\left(\alpha \right) & \left|x\right|\leq r_{xz}\cdot \cos\left(\beta \right) \end{array}$$

(6) $$\begin{array}{ccc} y_{\max}<r_{yz}\cdot \sin\left(\delta \right) & n<th_{n} & \left|y_{\max}-y_{\min}\right|<th_{s} \end{array}$$

Given $p={\left[\begin{array}{cccc} x & y & z & i \end{array}\right]}^{T}$ be a 3D point of the input point cloud, this point is filtered out from the next processing in case the location and the intensity criteria in Eq. (4) are not met. In this way, too saturated points in terms of intensity values or too far points are removed.

Some of the unwanted points due to the strong reflections of light on the floor are filtered out by using formulas in Eq. (5). By knowing the camera setting, i.e. the orientation α of the 3D camera with respect to the floor, it is possible to remove these unstable points in case these equations are not met.

The single cell analysis exploits Eq. (6) to discard outlier points but still preserving floating points related to hanging objects. In case one of these conditions is met, all the points belonging to the cell under investigation are removed.

Finally, the local neighbor filtering is applied to remove isolated bins of the occupancy grid. In case an active bin under investigation does not have a sufficient number of active neighbors (by looking at the 8-neighborhood of the investigated bin), this one is cleaned by all the 3D points inside to it. Please refer to Ref. [30] for further details about all these methods.

**Fig. 14:**
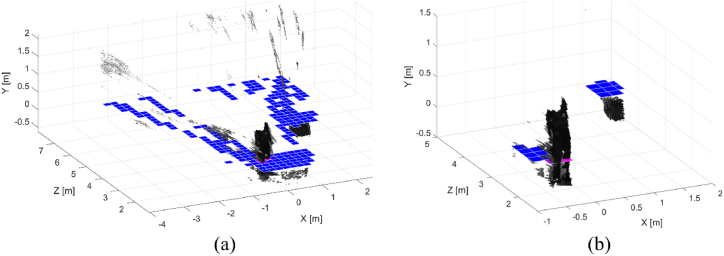
Input point cloud with the associated occupancy map before (a) and after (b) the cascade filtering. The two obstacles in the scene are correctly detected and reported in the occupancy map (in blue-pink tones).

After this procedure, the input point cloud (see Fig. 14(a)) is opportunely filtered in a way that the resulting point cloud and the related occupancy map contain only the actual objects, as shown in Fig. 14(b).

The two actual objects, i.e. the person and the box, are correctly detected. The computed occupancy map represents these obstacles by using blue-pink squares. Then, the obtained occupancy map is sent to the *navigation* module to attain obstacle avoidance by the AGV.

For the sake of completeness, the pseudocode of the developed methods is shown in Algorithm 3.

**Figure:**
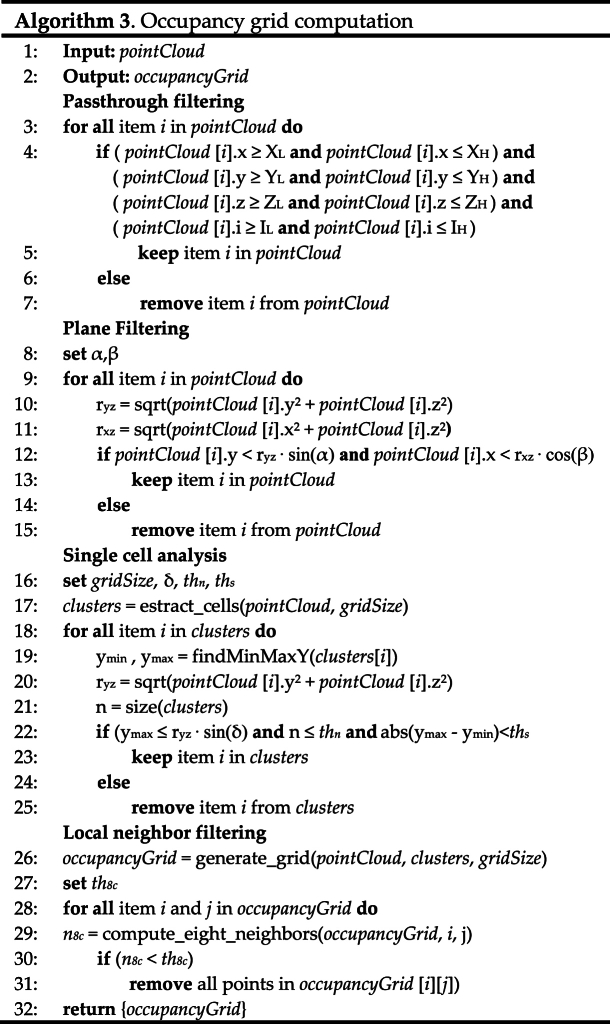

#### 3.2.2 Object detection

**Fig. 15:**
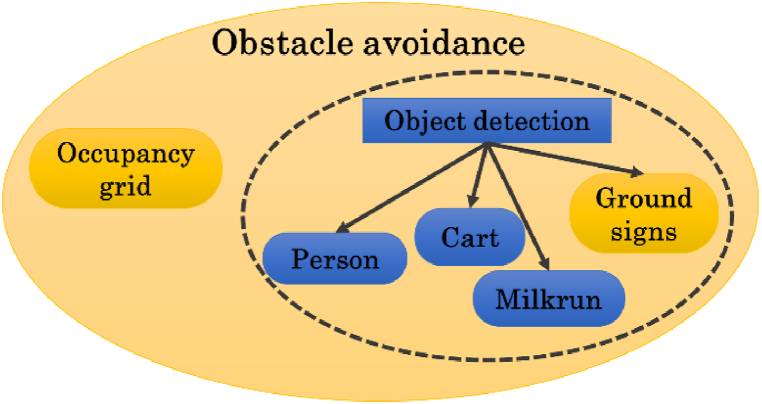
Sketch of the obstacle avoidance module that includes the object detection submodule. Three classes of objects are detected: person, cart, and milkrun.

The object detection module is in charge of detecting specific objects, such as people, milkruns, carts, and ground signs, to guide the AGV during the navigation. In this way, adaptive AGV motions can be planned according to the typology of the considered classes. Fig. 15 shows the sketch of the *obstacle avoidance* module, where the object detection module is marked in blue. In this case, three classes of objects are recognized: person, cart, and milkrun. Future activities will consider new other classes, e.g. ground sign, to introduce additional adaptive behaviors by the robot.

Some possible adaptive behaviors that will be implemented might be.

⁃ Stop the run when a person intersects the trajectory of the robot during the mission. Then, restart when the person goes out of sight or has changed direction.
⁃ When people are detected, try to stay as far away as possible from them to avoid fatal collisions.
⁃ In the presence of a milkrun that uses the same lane of the AGV, give priority to the milkrun. For example, the AGV can move to the side of the lane.
⁃ The robot cannot access restricted areas if the module detects sign delimiters.

To recognize the classes, a machine learning-based approach is adopted. As deep learning has achieved very impressive outcomes in solving significant challenges in many fields, including object recognition into images, we have used this approach for solving this task. Unlike traditional pattern recognition methods, which rely on human-designed features, deep learning automatically learns hierarchical feature representations from massive training data and extracts hidden input data factors through multi-level non-linear mappings [31].

**Fig. 16:**
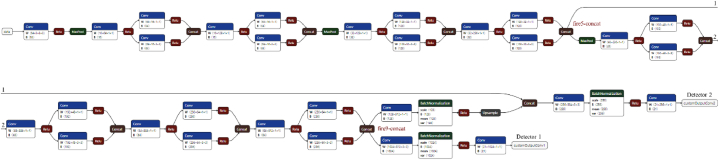
Custom neural network used for detecting the objects. It is composed of two detectors that examine the features extracted by SqueezeNet to detect objects.

The deployed network has been composed by using SqueezeNet [32] as backbone to extract the features. Then, two YOLO v3 detection heads [33] are added. In this regard, the size of the second detection head is twice the size of the first detector. This enables better identification of small objects in the scene at multiple scales. The first detection head uses the fire5-concat layer as source, whereas the second detection head uses the fire-9 concat layer, as shown in Fig. 16.

The input source is one color RGB image provided by the left camera of a stereocamera system. The input size is 227×227×3 pixels. The activation size of the first detector is 14×14×21, whereas the second detector has size 28×28×21. Six anchor boxes have been estimated to have better initial priors corresponding to the training data type, thus helping the detector to predict the boxes accurately during the learning phase. A supervised transfer learning approach has been considered for the network training. The loss function used for training is split into mean squared error for bounding box regression and binary cross-entropy for object classification.

**Fig. 17:**
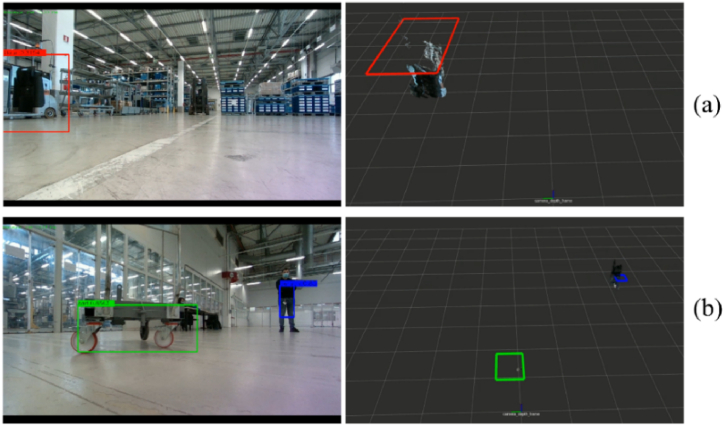
Examples of milkrun detection (a), and cart and person detection (b). Once the objects in the images are detected, their 3D reconstruction is performed by exploiting the depth map. Then, 3D occupancy maps are computed and sent to the navigation module for adaptive obstacle avoidance.

Once an object is recognized in the image under investigation by the custom network, the returned bounded box, which contains the information about the upper-left corner position, the width, the height, and the recognition score, is used to crop the depth map. In this way, the 3D reconstruction of the object can be obtained. Then, the 3D occupancy map can be easily computed and thus sent to the *navigation* module to perform adaptive obstacle avoidance. In Fig. 17, two examples of recognition by the network are reported. The milkrun is detected in Fig. 17(a), whereas one person and one cart are identified by the network in Fig. 17(b). Additional quantitative details can be found in Section 4.

**Fig. 18:**
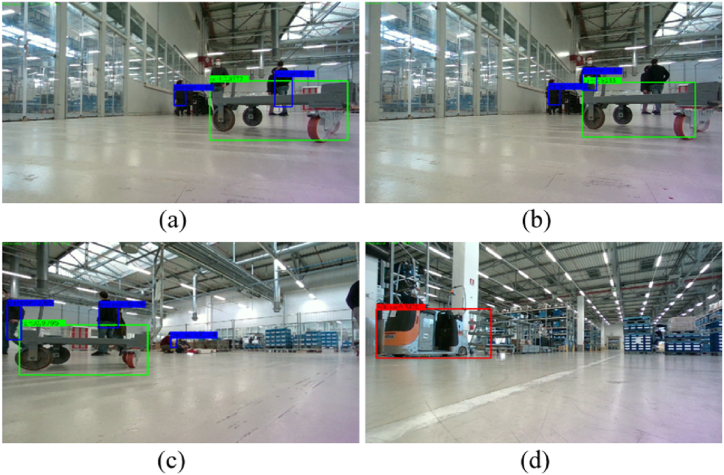
Some examples of detection fails due to partial occlusions and overlapping of objects to be recognized. Some people behind the cart are not well recognized such as in (a,b,c). The person in (d) is partially covered by the milkrun, thus hampering the correct detection.

Although the good performance by the proposed architecture in recognizing these specific objects, it still requires improvements in future activities. Specifically, in case of occlusions or overlapping objects, the network cannot always provide accurate recognition. The people behind the cart are not consistently recognized by looking at Fig. 18(a)-(b). Moreover, in Fig. 18(b), the two near people are detected as only one person. In Fig. 18(c), two out of the three people behind the cart are detected. Also in this case, the two people are very close to each other, thus hindering the correct detection. Another example of partial occlusion is reported in Fig. 18(d), where the person is located behind the milkrun. This aspect represents a limitation of the presented network to be addressed in future developments.

### 3.3 Tag detection module

**Fig. 19:**
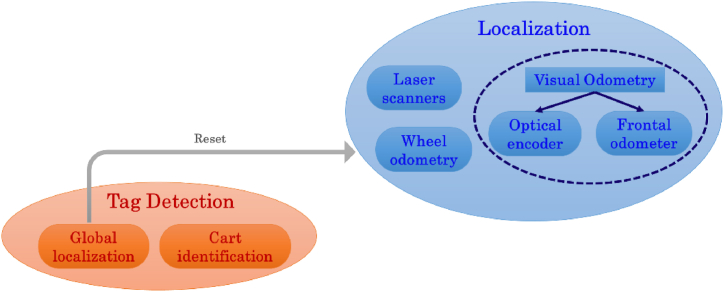
Scheme of the tag detection module. The global localization module sends the resetting signals to the localization module when it recognizes specific tags. The cart identification module recognizes the cart tags.

The *tag detection* module recognizes fiducial markers, also called tags. Some tags are reserved for global localization purposes, whereas others are used for cart identification. When a tag for global localization is recognized, a reset signal is sent to the visual odometry and wheel odometry modules of localization in order to reset the cumulating drift errors. By knowing the pose of the tag in the global map, it is possible to estimate the relative pose of AGV with respect to this tag and thus reset all the odometry systems. A scheme of the tag detection module is reported in Fig. 19.

**Fig. 20:**
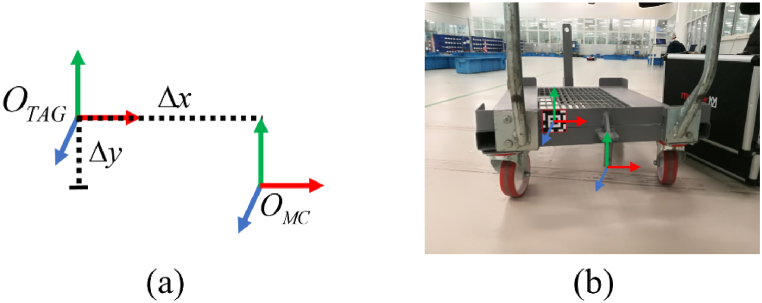
(a) Sketch of reference systems for the tag and the hooking point of a cart. (b) Reference systems superimposed over an actual cart.

The cart identification module can recognize tags fastened on the carts. This module aims to return the information of the middle point necessary for centering the AGV under the cart during the picking phase. The location of this middle point is known at priori after having fixed the tag on the cart, as shown in Fig. 20. Assuming that the tag reference system $O_{TAG}$ is located at the middle of the tag, and the middle cart reference system $O_{MC}$ at the hooking point (see Fig. 20(a)), it is possible to measure for each cart the x- and y-displacements Δx and Δy. A list of tags, containing the Identification number ID and the displacement data, can be stored in the *tag detection* module. When the module recognizes a registered ID tag, the pose of the middle point of cart can be estimated in the 3D space. The AGV can use this information for centering itself before picking the cart. Fig. 20(b) shows the reference systems superimposed over a cart.

The cart docking is attained by using two coarse-to-fine complementary mechanisms. The first method, i.e. the one described in this section, provides an initial coarse alignment of the AGV with respect to the middle point of the cart by using vision. The second approach exploits the 2D lidar signals and proximity sensors to detect the cart finely. When the tag placed on the cart is not visible anymore by the camera due to the short distances between the AGV and the cart itself, the second mechanism provides the information to accomplish the docking phase according to a closed-loop control.

## 4 Results

This section reports qualitative and quantitative results related to the previously described modules. The effectiveness and robustness of the proposed methods have been evaluated through several experiments. The downward-looking vision-based odometer (DW-L odometer) comprises a 2D grayscale camera of resolution 608×808 pixels with a pixel size of 4.8 μm [34]. A lens [35] having a focal length of 3.6 mm completes the acquisition system. The forward-looking vision-based odometer (FW-L odometer) comprises the stereocamera Intel RealSense D455 [36], having a baseline of 95 mm and an operating range from 0.4 m to 6 m. The S-BL stereocameras (please refer to Fig. 3) are Intel RealSense D435 sensors, having a baseline of 50 mm with an operating range of 0.3–3 m. Two separate processing units are used for estimating the information of interest. In detail, the visual odometry module uses the NVIDIA Jetson AGX Xavier, having one 512-core Volta GPU, for processing the images directly on GPU. The Intel NUC 9 [37] is used by the *obstacle avoidance* module and the *tag detection* module for providing other information of interest. The processing methods have been coded using Python and C++. Frameworks and libraries such as OpenCV [38] and PCL (Point Cloud Libraries) [39] have been used. The information of interest between the robot and the modules is exchanged using ROS (robot operating system) [40].

The following subsections describe in detail the qualitative and quantitative results of the described modules.

### 4.1 Visual odometry outcomes

**Fig. 21:**
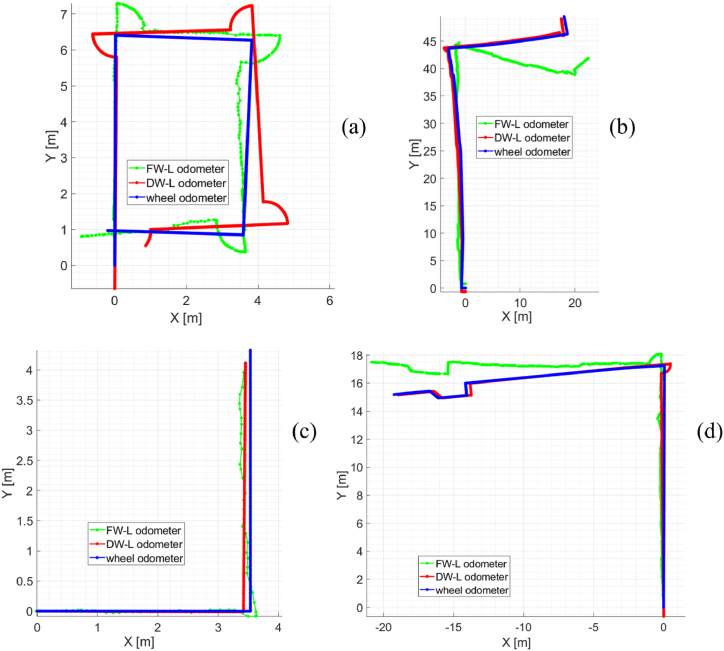
Trajectories performed in the industrial plant. In (c), only pure translations have been attained. Arches represent pure rotational movements on the AGV barycentre as in (a)-(b)-(d).

The outcomes related to the visual odometry module are reported here. The module provides the relative and absolute pose of the robot estimated by exploiting 2D/3D images. Different trajectories have been performed. The information returned by the wheel odometry has been used as ground truth to validate the method. Some trajectories are shown in Fig. 21. As one can note, the DW-L odometer (downward-looking vision-based odometer) seems to be more accurate than the FW-L odometer (forward-looking vision-based odometer) by qualitatively comparing the signals with the reference one provided by the wheel odometer. This is likely due to the intrinsic accuracy of the used sensors. Moreover, downward-looking cameras are far less likely to capture noise in the form of motion of external entities when compared to forward-looking ones. In this regard, the FW-L odometer uses 2D and 3D data to estimate the motion. However, motion estimation mainly depends on the accuracy of 3D data. In fact, the higher is the distance between an object and the sensor, the higher could be the error in the 3D reconstruction. As already described, the 3D location of 2D keypoints detected over consecutive images can be computed. If the 3D data is initially inaccurate, also the pose estimation might be intrinsically affected. In this regard, the relative error along the Z-axis of the used stereocamera is 2%, as from the datasheet. This aspect is also found by observing the results reported in Table 2. In most tests, the FW-L odometer performs worse than the DW-L odometer. In acquisition III (see Fig. 21(c)), lower positioning errors (see Table 2) are obtained by the FW-L odometer. This might be due to the fact that only pure translations have been performed in this test. Higher heading RMS (root mean squared) errors can be found in the other tests, which also contain rotations.

**Table 2:** Quantitative results for the tests are shown in Fig. 21. The values in bold are the best for each acquisition.

Acquisition	Odometer type	Positioning RMSE (m)	Heading RMSE (Deg)	Final positioning error		Final heading error		Covered distance (m)
				(m)	(%)	(Deg)	(%)	
*I* Fig. 21(a)	FW-L	0.85	7.56	2.21	37.36	38.91	44.35	26.74
	DW-L	**0.91**	**2.61**	**0.89**	**21.59**	**7.01**	**7.98**	23.67
*II* Fig. 21(b)	FW-L	3.37	19.56	9.55	7.78	70.18	69.49	75.19
	DW-L	**0.56**	**3.99**	**0.63**	**0.68**	**4.03**	**3.99**	69.29
*III* Fig. 21(c)	FW-L	**0.25**	1.02	0.39	**0.88**	1.71	1.90	7.79
	DW-L	0.26	**0.02**	**0.23**	4.05	**0.01**	**0.003**	7.55
*IV* Fig. 21(d)	FW-L	1.87	7.78	3.25	8.83	12.05	6.50	41.12
	DW-L	**0.26**	**0.37**	**0.34**	**0.68**	**0.56**	**0.30**	38.04

Being the final pose estimated by cumulating the previous motions, errors in some of the relative pose estimates can negatively affect the final reconstruction. Consequently, the localization module exploits the periodic signals from the *tag detection* module to reset the odometers from unavoidable drift errors. Integrating different signals returned by multisensorial systems helps to manage critical situations (e.g. one of the sensors is not providing useful information).

Overall, average Positioning RMSE values of 1.59 m and 0.49 m are found for the FW-L odometer and DW-L odometer, respectively. The corresponding heading RMSE values are 8.98 Deg and 1.75 Deg. These results prove that the proposed visual odometry-based systems are valid for estimating the robot pose in the industrial environment, thus meeting the requirements and the specifications of the research project.

### 4.2 Obstacle avoidance outcomes

**Fig. 22:**
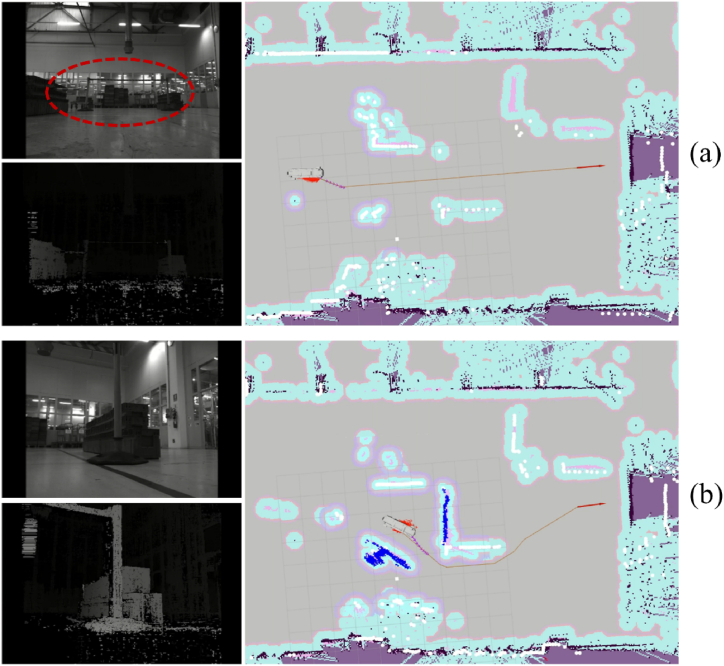
(a) the planner module sets the mission. The vision systems detect the hanging obstacle in the red mark during the mission (b). The laser scanners cannot detect floating obstacles. The occupancy grids returned by the vision are represented in blue tones, whereas the obstacles detected by the laser scanners are white dots. Also, the depth images are reported below the left images of the frontal stereocamera.

Different tests have been run to validate the developed modules for obstacle avoidance. By taking a look at Fig. 22(a)–a mission starting from left to right of the area has been created (see the red path in the map). The AGV uses both the vision systems and the laser scanners from Hokuyo [41] (having a 2D field of view of 270 Deg), to detect obstacles. However, the 2D laser scanners cannot detect floating or hanging obstacles like those in the red mark. Initially, the stereocamera cannot detect the hanging obstacle due to the limited operating range. After the mission started, the vision module also perceived the presence of the hanging obstacles (see the blue points in Fig. 22(b)). Consequently, the trajectory has been replanned to overcome the obstacle and reach the ending position on the map beyond the obstacles.

The occupancy maps of the surrounding environment are computed by the vision systems and used to avoid all types of obstacles. The navigation module can replan the trajectories according to the position of the obstacles.

**Fig. 23:**
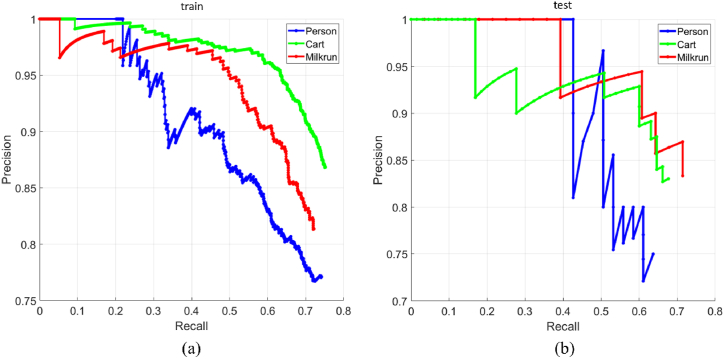
Precision-recall curves for the (a) training set and the (b) testing set.

Besides, when the object detection module recognizes people, carts, and milkruns, the 3D location is returned to the navigation module, which implements the logics of avoidance described in Section 3.2.2. The custom CNN-based network in charge of recognizing these objects has been trained using a supervised transfer learning approach. A total number of 6723 images have been labeled. The 80% of the entire dataset has been dedicated to the training set (5378 images), whereas the remaining 20% is used to define the testing set (1345 images). The dataset contains 2879 people, 6487 carts, and 3817 milkruns. To have an idea of the training quality, the precision-recall curves are computed for the training set (Fig. 23(a)) and the testing set (Fig. 23(b)).

A high area under the curve precision-recall stands for both high precision and high recall. High precision is related to a low false positive rate, whereas high recall relates to a low false negative rate.

The curves in Fig. 23 show that the recall values are lower than the precision ones. This means that a high false negative rate is obtained, i.e. very few results are returned, but most of the predicted labels are correct. The precision P and recall R are defined in Eq. (7).

(7) $$\begin{array}{c} P=\frac{T_{P}}{T_{P}+F_{P}}\\ R=\frac{T_{P}}{T_{P}+F_{N}} \end{array}$$

The $T_{P}$, $F_{P}$, and $F_{N}$ represent the number of true positives, false positives, and false negatives, respectively.

The average class precision values for the training set of the three classes are 0.61 (person), 0.73 (cart), and 0.68 (milkrun), respectively. The average class precision values for the testing set are 0.59 (person), 0.64 (cart), and 0.69 (milkrun). These results show that the class person has the lowest precision values. This is probably due to a slight unbalancing of classes. In fact, the class person has the lowest number of instances. However, this aspect does not hinder the object detection module that still ensures high recognition scores. Another valuable metric for evaluating the performance of the object detector is the IoU (Intersection over Union) score. Given two bounding boxes (one is the predicted and the other one is the ground truth), the IoU is defined as the area of overlap between them over the area of union. The IoU values for the training set are 0.65 (person), 0.69 (cart), and 0.69 (milkrun). Besides, the IoU values for the testing set are 0.64 (person), 0.69 (cart), and 0.65 (milkrun). These results confirm that the trained net is suitable for detecting these object classes, ensuring high accuracy. All the quantitative outcomes are summarized in Table 3.

**Table 3:** Statistics related to the CNN-based network trained to recognize specific object classes.

Class name	Training set		Testing set		
	Average class precision	IoU	Average class precision	IoU	
Person	0.61	0.65	0.59	0.64	
Cart	0.73	0.69	0.64	0.69	
Milkrun	0.68	0.69	0.69	0.65	
	**0.67**	**0.68**	**0.64**	**0.66**	**Average**

### 4.3 Tag detection outcomes

**Fig. 24:**
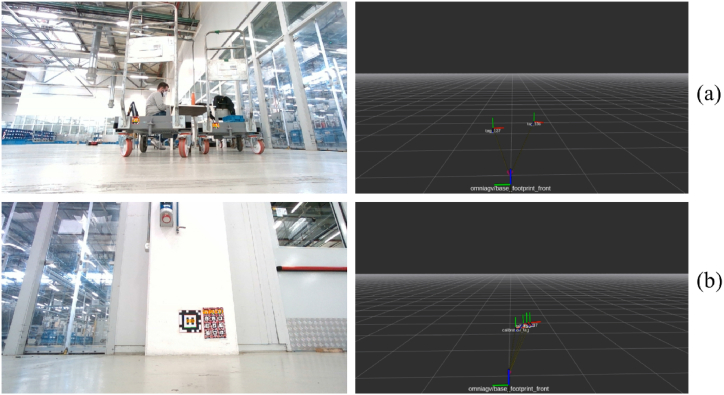
(a) two tags associated with two different carts are detected. Their 3D poses are reported on the right. (b) One tag for absolute pose estimation is detected together with the other three tags registered for cart identification.

The tag detection module has a twofold task: provide the global pose of the robot and identify the middle of one cart for enabling cart docking. For these purposes, fiducial markers belonging to the AprilTag 2 family [42] have been used. By observing Fig. 24(b), the wider tag, having a size of 21.6 cm, has been deployed to estimate the global pose of the robot. This type of tag has been fastened to specific points in the plant where the AGV works. When one of these tags is recognized by our module, the absolute pose of the robot is derived. This way, the odometry systems affected by drift errors can be reset.

Smaller tags, such as the ones in Fig. 24(b), are placed on the carts. The size of this tag is 6.4 cm. By knowing the position of the tag on the cart, the 3D cart's middle point can be easily estimated. This information is then used to help center the AGV under the cart during the picking.

The 3D poses of the tags in the world are represented on the right of Fig. 24. In Fig. 24(a), two carts are identified, whereas in Fig. 24(b), one tag for global positioning and three tags for cart identification are recognized.

In order to have a quantitative evaluation of how the tag pose affects the recognition phase, different experiments have been run. The experimental setup includes.

• Intel Realsense D455 camera (images acquired from the RGB camera at a resolution of 1280 × 720 px)
• Square Apriltag tag (*tagStandard41h12* family, size 100 × 100 mm)

The size of the Apriltag is compatible with the ones used in the actual case study. The tag is acquired from frontal and perpendicular perspectives to the acquiring sensor, even when lying on the floor. All the experiments carried out, including the results averaged over ten acquisitions per experiment, are reported in Table 4. The height of the sensor to the ground is 24 cm.

**Table 4:** Recap of the experiments. In the Note column, the letter F stands for Frontal Tag, the P means Perpendicular tag, while FLO refers to the floor.

ID	TARGET DISTANCE [m]	Note	Translation (mean and std. dev.) [m]	Rotation (mean and std. dev.) [deg]
00	1.50	F	[-0.0273, 0.1289, 1.4597] [0.0007, 0.0029, 0.0336]	[-2.9544, 9.1783, 71.5984] [9.8486, 6.9709, 4.5038]
01	1.00	F	[-0.1086, 0.1064, 0.9928] [0.0005, 0.0006, 0.0052]	[22.5438, 1.8630, 178.6620] [2.1468, 2.5724, 0.6190]
02	0.50	F	[-0.1114, 0.0836, 0.5200] [0.0003, 0.0002, 0.0013]	[5.3382, −1.4180, 178.7600] [0.7626, 1.0505, 0.1275]
03	1.50	P	[0.8122, 0.1471, 1.7141] [0.1034, 0.0189, 0.2182]	[62.5805, −55.7093, 146.6080] [16.9505, 11.5508, 17.2701]
04	1.00	P	[0.4509, 0.1092, 1.0454] [0.0280, 0.0068, 0.0649]	[43.5007, −78.5517, 72.4681] [9.1887, 5.6280, 7.3190]
05	0.50	P	[-0.2131, 0.0781, 0.4699] [0.0038, 0.0014, 0.0084]	[-108.2040, 83.7158, 78.5070] [6.8650, 1.3122, 7.5507]
06	Minimum detection (∼0.50)	FLO	[-0.1013, 0.2423, 0.4738] [0.0012, 0.0029, 0.0056]	[93.7794, −7.2092, 171.8680] [0.4882, 0.7817, 0.7064]
07	Maximum detection (∼0.90)	FLO	[0.1193, 0.3116, 0.9647] [0.0071, 0.0183, 0.0565]	[-88.9813, 6.3549, −4.6825] [2.1766, 8.7875, 5.5388]

The experiments show how the target recognition occurs easily under controlled conditions. The Intel Realsense D455 color camera can recognize the Apriltag tag in a series of configurations, but it has been experimentally verified that its recognition performance could be affected by reflections or overexposures of the image where the tag is depicted. The measurements of translation and rotation are relatively stable (the standard deviation values are low) at distances ranging from 0.5 m to 1.5 m (compatible with the docking requirements), but show some limitations for the accuracy of localization on the *Z*-axis (distance from the sensor) due to the estimation of the position of the tag itself. However, considering the specific requirements described beforehand, the omnidirectional robot can easily approach the goal by combining translations and rotations in the docking procedure. For this reason, the detection accuracy in estimating the tag pose with respect to the robot observer is acceptable and in line with the requirements.

## 5 Conclusions and future works

In this article, an omnidirectional automated guided robot (also called as omniagv) devised for material transportation in the manufacturing industry has been presented. The particularity of the environment where the robot navigates has led to the development of different robust and accurate vision-based modules. In this regard, the very low-textured surfaces of the settings, the presence of moving obstacles (such as people, milkruns, etc.), and the requirement of avoiding expensive changes to the facilities, have brought to devise effective and reliable methods for the adaptive robot navigation and localization in this critical use case. The navigation module comprises three submodules: localization, obstacle avoidance, and tag detection. Each module exploits the vision perception to provide helpful information for safe and accurate navigation. The localization module uses wheel odometry, visual odometry, and laser scanners for deriving the relative robot pose in the environment. The obstacle avoidance module detects moving and stationary obstacles besides recognizing some object classes for implementing adaptive navigation behaviors. Finally, the tag detection module aids the AGV in hooking the carts and provides resetting signals to the localization module when specific fiducial markers are recognized by vision.

An extensive quantitative and qualitative validation of the proposed methods has been provided through controlled indoor tests, according to the use cases and the purposes of the research project. Good performance in terms of the robustness and effectiveness of the presented solution has been achieved.

In designing, implementing, and then testing any system, a few ideas for further improvements are always bound to present themselves.

One of the advancements reported here is the design and implementation of a downward-facing optical encoder. In doing that, a custom squared 3D printed solution hosting both the lighting system and the vision sensor is presented. A squared design is a natural choice for a vision system devised for a holonomic robot, capable of moving in any direction on the spot, including "horizontal” movements. However, the AGV is used for docking and moving carts, which have a rectangular shape and their physical properties, especially inertia and center of mass when not empty, thus affecting the whole mechanical stability of the robot while moving in different directions. The navigation sub-system takes care of this by restraining to slower movements when faster ones could be dangerous. Considering this additional system constraint, it might be beneficial to investigate whether the downward-facing optical encoder can be redesigned to consider such an asymmetry in practical usage terms.

Localization and navigation can also be enhanced by further integration between individual subsystems, where object detection and occupancy grid modules can be used for a better estimation of the way the robot is moving. In particular, the robot has to “share” the floor with other moving entities, be them other robots of the fleet, people, and old-fashioned milkruns. Their detection and representation in the occupancy grid can be used to provide feedback on this information to the forward-looking cameras, contributing to a better estimation of the robot locomotion, filtered of external noise induced by other moving entities. This might contribute to enhancing the accuracy of the forward-facing cameras, which experiments have shown to be lacking, especially when compared to the downward-facing optical encoder performance.

Future activities will also be led to enhance the capability of the AGV in recognizing other specific objects (e.g., floor signs, area delimiters, and so on) to implement further adaptive behaviors in the presence of these object categories. For example, floor signs are frequently used in real-world scenarios to signal wet or dirty floors, affecting the way factory workers navigate in the environment. Handling such cases can further enhance the usability of AGV in the real world.

In addition, a vision-based system for extracting natural landmarks into the environment and better recognition of carts will also be developed. In particular, the docking solution currently employs visual markers (Apriltag) and laser scanners. The solution is quite effective and limited to particular objects (carts) that can be loaded and unloaded in specific areas. This helps not only in enabling safe and precise docking operations but also in a periodic reset of odometry-based systems present where every mission starts or ends. Extracting natural, stable, markers from the environment can further reduce the need for “altering” the working environment by harnessing the information that is already there. At the same time, 3D and color cameras, designed for mobile and embedded systems, will still evolve, and this will undoubtedly pave the way for detecting and better localizing carts in relation to the AGV just based on their shape and appearance, with benefits in the enhancement of docking operations.

## Funding

This research was funded by the project OMNIAGV4.0, grant number X7H8LZ3 - POR Puglia FESR-FSE Azione 1.6 "Innonetwork" A.D n. 34 April 11, 2018 and A.D. n. 41 April 20, 2018.

## Data availability statement

Data will be made available upon private request to the authors.

## CRediT authorship contribution statement

**Cosimo Patruno:** Writing – review & editing, Writing – original draft, Validation, Software, Methodology, Investigation, Data curation, Conceptualization. **Vito Renò:** Writing – review & editing, Validation, Project administration, Methodology. **Massimiliano Nitti:** Visualization, Validation, Software. **Nicola Mosca:** Writing – review & editing, Formal analysis, Data curation. **Maria di Summa:** Writing – review & editing, Data curation. **Ettore Stella:** Supervision, Resources, Project administration, Funding acquisition, Conceptualization.

## Declaration of competing interest

The authors declare the following financial interests/personal relationships which may be considered as potential competing interests:

Ettore Stella reports financial support was provided by Puglia Region.
